# Episodic Visual Hallucinations, Inference and Free Energy

**DOI:** 10.3390/e26070557

**Published:** 2024-06-28

**Authors:** Daniel Collerton, Ichiro Tsuda, Shigetoshi Nara

**Affiliations:** 1 Faculty of Medical Sciences, Newcastle University, Newcastle upon Tyne NE1 7RU, UK; 2 AIT Center, Sapporo City University, Sapporo 005-0864, Japan; i.tsuda@scu.ac.jp; 3 Graduate School of Environmental, Life, Natural Science and Technology, Electrical and Electronic Engineering Department, Okayama University, Okayama 700-8530, Japan; nara@ec.okayama-u.ac.jp

**Keywords:** Active Inference, generative inference, free energy, visual hallucination, discriminative inference, phase transition

## Abstract

Understandings of how visual hallucinations appear have been highly influenced by generative approaches, in particular Friston’s Active Inference conceptualization. Their core proposition is that these phenomena occur when hallucinatory expectations outweigh actual sensory data. This imbalance occurs as the brain seeks to minimize informational free energy, a measure of the distance between predicted and actual sensory data in a stationary open system. We review this approach in the light of old and new information on the role of environmental factors in episodic hallucinations. In particular, we highlight the possible relationship of specific visual triggers to the onset and offset of some episodes. We use an analogy from phase transitions in physics to explore factors which might account for intermittent shifts between veridical and hallucinatory vision. In these triggered forms of hallucinations, we suggest that there is a transient disturbance in the normal one-to-one correspondence between a real object and the counterpart perception such that this correspondence becomes between the real object and a hallucination. Generative models propose that a lack of information transfer from the environment to the brain is one of the key features of hallucinations. In contrast, we submit that specific information transfer is required at onset and offset in these cases. We propose that this transient one-to-one correspondence between environment and hallucination is mediated more by aberrant discriminative than by generative inference. Discriminative inference can be conceptualized as a process for maximizing shared information between the environment and perception within a self-organizing nonstationary system. We suggest that generative inference plays the greater role in established hallucinations and in the persistence of individual hallucinatory episodes. We further explore whether thermodynamic free energy may be an additional factor in why hallucinations are temporary. Future empirical research could productively concentrate on three areas. Firstly, subjective perceptual changes and parallel variations in brain function during specific transitions between veridical and hallucinatory vision to inform models of how episodes occur. Secondly, systematic investigation of the links between environment and hallucination episodes to probe the role of information transfer in triggering transitions between veridical and hallucinatory vision. Finally, changes in hallucinatory episodes over time to elucidate the role of learning on phenomenology. These empirical data will allow the potential roles of different forms of inference in the stages of hallucinatory episodes to be elucidated.

## 1. Introduction

In addition to his substantial contributions in many fields of neuroscience, Karl Friston has been one of the key theorists on the mechanisms underlying visual hallucinations. His 2005 commentary, “Hallucinations and perceptual inference” [1] on Collerton et al.’s Target Article “Why people see things that are not there” [2] was the first explicit application of generative approaches underpinned by mechanisms to minimize free energy to understand visual hallucinations. He has continued this active participation through to the 2023 publication of an extensive review of existing models and a new integrated theoretical framework for modelling hallucinations [3]. It is therefore a great pleasure to participate in this Special Issue of *Entropy* to mark his 65th birthday.

We have taken the opportunity to give a personal perspective on how generative and other mechanisms have influenced the understanding of hallucinations over the last twenty years, to highlight some current areas where data and theory are lacking, and to suggest fruitful avenues of research for the future.

### 1.1. The Importance of Visual Hallucinations

Visual hallucinations come in a variety of different forms and occur in many different people. In this paper, we will focus on complex, sometimes called formed, hallucinations of meaningful things, usually of people, faces, animals, or objects [2,3]. These experiences can be an infrequent feature of normal life for many people. However, they are predominantly associated with three groups of clinical disorders: Lewy body disorders such as Parkinson’s disease, visual impairment due to various eye diseases, and psychotic disorders such as schizophrenia. They are often a clinical marker of increased disease severity and greater personal impact. Hence, understanding their genesis in order to improve treatments is an area of active investigation.

Hallucinations are a variation on veridical perception. Veridical perception depends upon the synchronized function of multiple interacting sensory and cognitive systems [4]. Each is functionally specialized and acts at different levels and timescales. Though not always error-free, the output of these systems in terms of an object perception is remarkably consistent. Hence, veridical vision can be characterized as a one-to-one correspondence between what is perceived and what is out there, with the added property that this correspondence is consistent from one person to another: if I see a dog, other people will also see a dog. 

Visual hallucinations, in contrast, are defined as seeing something that is not there [2,3]. Thus, the usual correspondence is lost. If I and most others see an empty room, the hallucinator sees a person in that room that neither I nor those others can see. (They may also have the additional property that this corresponding hallucination is unique to that individual, though this is not established given the rareness of people hallucinating alongside each other.)

**Figure 1:**
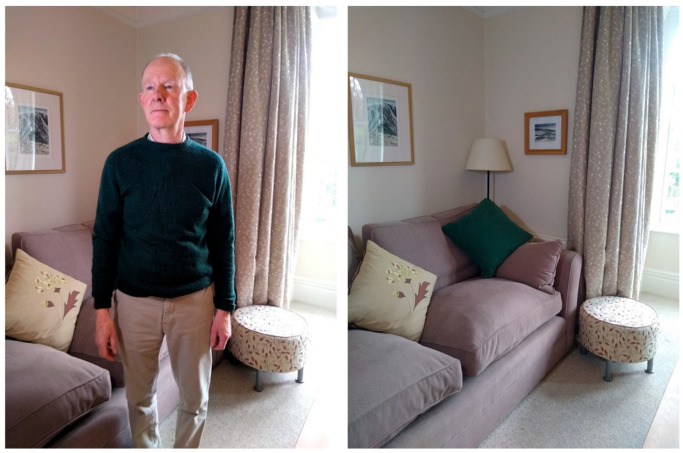
Reconstruction of a hallucination of a person in a room. To see the figure that is not there is also to fail to see the lamp and green cushion that are there.

An under-recognized corollary of this definition is that hallucinations are equally characterized by not seeing something that is there. Thus, as Figure 1 illustrates, a hallucination is both to perceive an incorrect object and to fail to perceive the correct object. 

### 1.2. Generative Models of Visual Perception

**Figure 2:**
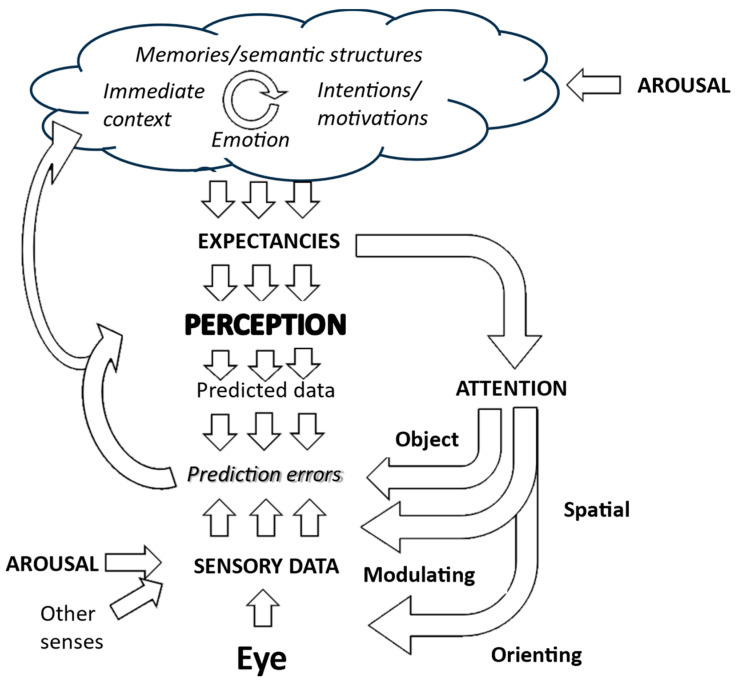
Generative model of visual perception. The perception is driven by expectations which are created by a complex interaction of higher-level cognitive functions. Predictions of incoming sensory data drawn from that perception are then compared with actual sensory data, with the perception then being modified to minimize any mismatch. Expectations also drive attention and action to focus the gathering of sensory data on areas that are most relevant to testing predictions. Further details are in Collerton et al., 2023 [3].

This corollary is particularly relevant for generative models of perception, such as those that provide the foundation for Friston and colleagues’ Active Inference account of visual hallucinations [3]. Generative approaches propose that subjective visual and other perceptions map onto internal representations of the world. These representations are continually transformed to minimize any mismatch between predictions of sensory data derived from them and actual sensory data. Hence, they are updated to reflect what is actually there (Figure 2). 

Since generative models are not directly derived from sensory data but from internal predictions of what should be seen, they always have the potential to see something that is not there—to hallucinate. It is the rapid, accurate adjustment of the internal model in response to mismatches between what is expected to be seen and what is there to be seen that keeps them veridical. Hence, perception remains swift and accurate even if perceptions are unpredictable, for example, when watching a film or television program in which there are quick discontinuous changes in scenes and objects.

### 1.3. Active Inference and Hallucinations

As applied to hallucinations, Active Inference therefore combined two factors to explain why hallucinations occur. Firstly, there is an internal representation of the world which contains the hallucinated perception—an expectation of seeing the thing that is not there. Secondly, there is a failure to adapt that representation in the absence of corresponding sensory information—an inability to see the thing that is there. Computationally, there are two factors. Either an over-confident prior belief in the hallucinatory perception is given too high a weight in computations, and/or sensory data are given too low a weight. In the latter case, data may be absent. Alternatively, they may be ignored if present because they are predicted to be so imprecise as to be irrelevant.

### 1.4. The Challenge of Episodic Hallucinations

**Figure 3:**
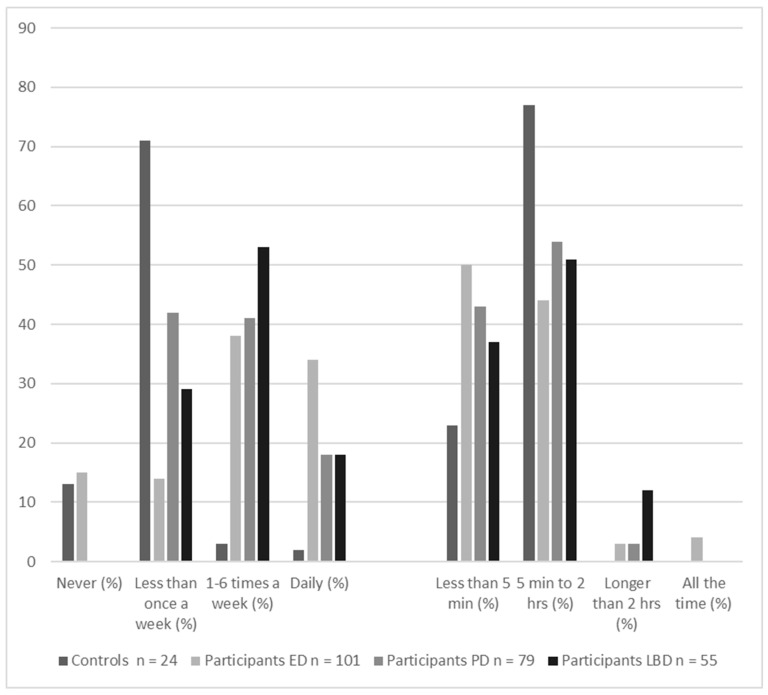
Frequency and duration of the visual hallucinations reported in the previous month by controls (reported by 15% of sample) and three at-risk groups: participants with eye disease (ED, 75% of sample), participants with Parkinson’s disease (PD, 51% of sample), and participants with Lewy body disease (LBD, 70% of sample). The majority of participants in all groups had hallucinations less than daily for less than two hours. Data taken from Urwyler et al., 2016 [5].

Recently, there has been an upsurge of interest in why hallucinations do not happen more frequently. Most of the factors in all models are consistently present, so why are hallucinations not? As Figure 3 shows, visual hallucinations are episodic. Even in people who are prone to hallucinations, most visual perception is veridical. If it is incorrect, the error lies in not recognizing what is there—agnosia rather than hallucinations. That is, it is the corollary to but not the proposition that a hallucination is seeing something that is not there. Even in the 4% of people with eye disease who report continual hallucinations, individual hallucinations are temporary. 

As applied to Active Inference, why are priors only occasionally overly precise, and why are sensory data only sometimes under-precise or absent? 

### 1.5. Biases in the Data on How Hallucinations Occur Only Sometimes

In trying to answer this question, it is striking that the overwhelming majority of research on visual hallucinations over the last two decades has focused on the people who experience them. Of the ten most highly cited non-review papers on visual hallucinations on Web of Science [6,7,8,9,10,11,12,13,14,15], three were purely observational and evaluated the characteristics of people who hallucinated, five compared people who hallucinate with similar clinical states who do not, and two compared people when they were and were not hallucinating. Even looking at less influential papers, there have been no publications which have tracked transitions from veridical to hallucinatory vision, and none which have systematically looked at links between the environment and hallucinations.

**Figure 4:**
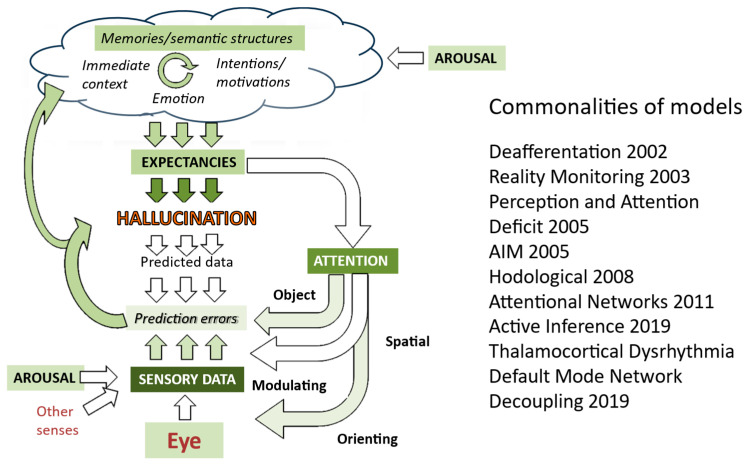
Mapping of models of hallucinations developed over the last two decades onto aspects of visual systems (derived from Collerton et al., 2023 [3]). Depth of color indicates the number of approaches which highlight a specific aspect, with darker green indicating a greater number of models. Thus, sensory data, attention, and expectancies are common to most models. Further details of each model are in Collerton et al., 2023.

Models of how hallucinations occur are therefore almost exclusively based upon what happens in the brains of people who have hallucinations. The most recent summary of models (Figure 4) illustrates this blind spot for external factors which might account for why hallucinations occur episodically—no approaches explicitly include environmental factors.

An analogy can help explore why comparing hallucinating and no-hallucinating states has intrinsic limitations. We can consider a hallucinatory image as a transient state of the perceptual system. The movement from veridical to hallucinatory perception is therefore a transition from one state to another.

**Figure 5:**
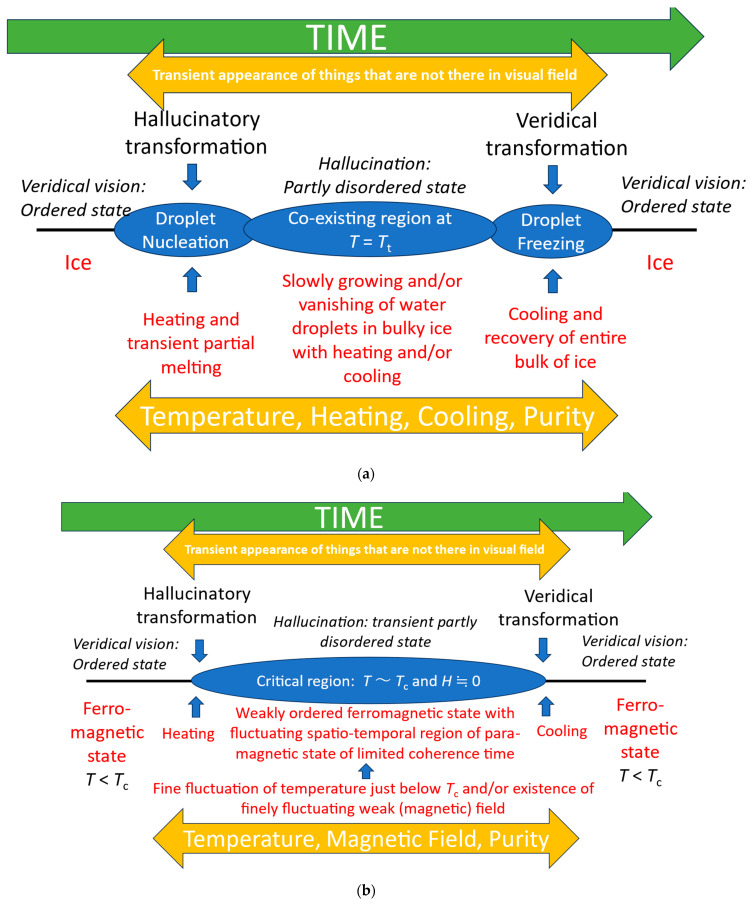
(**a**) Illustration of the correspondence between features of episodes of visual hallucinations and first-order phase transitions. We use the analogy that veridical perception is akin to a stable solid such as ice—there is a consistent ordered one-to-one mapping between object and perception—and that fully hallucinatory vision is sufficiently distinct from fully veridical vision to be analogous to a different state, akin to water. However, panoramic hallucinations in which veridical vision is entirely replaced by a hallucinatory scene are rare [20], and are only reported in eye disease. Most hallucinations, including those in eye disease, are of a hallucinatory element within a veridical scene as illustrated in Figure 1. In these cases, a partially disordered mixed-phase regime of solid (veridical) and liquid droplets (hallucinations) is a better analogy. *T*—temperature; *T_t_*—transition temperature. (**b**) Illustration of the correspondence between features of episodes of visual hallucinations and the second-order phase transition between ferromagnetic and paramagnetic states [17,18]. *T*—temperature; *T_c_*—critical (Curie) temperature; and *H*—strength of magnetic field.

In his 2007 paper [16], Friston states that organisms aim to maintain homeostasis by avoiding physical first-order phase transitions such as those between solids and liquids. We suggest that there is a similar principle is at work within brain systems. Organisms seek to maintain a stationary state of perception in which objects in the environment have a consistent unique mapping onto internal representations. We propose that hallucinatory perception is a qualitatively different state which transitions from this stationary mapping to a nonstationary transitory idiosyncratic mapping during hallucinatory episodes (see legend to Figure 5 for more details). 

Looking for inspiration from other systems which exhibit qualitative changes suggests that physical phase transitions may be a useful heuristic [17,18]. Similar analogies have been used in conceptualizing the transition from walking to running [19]. In particular, we can consider the hallucinatory phase as an informational transition from a stable veridical phase, to a mixed-phase regime [Figure 5] which, as Figure 1 illustrates, retains aspects of veridical perception along with hallucinatory elements.

Four aspects of physical phase transitions highlight areas where current data are lacking.

Firstly, these transitions only occur for a relatively restricted set of values for relevant variables. Investigating variables outside of that range is not productive. To take our melting analogy in Figure 5a, temperature comparisons above or below melting point tell us nothing about the specific temperature at which partial melting occurs. At present, research designs have achieved the equivalent of comparing locations (people) in which ice (veridical perception) is common with locations (people) where liquid water (hallucination) is common. Though differences between a fridge and a freezer can suggest factors which influence the occurrence of ice, they provide little insight into the process by which ice transforms into water. Comparing a freezer when it is turned on and turned off (people when they are not or are hallucinating) gives greater specificity in identifying relevant variables to consider in the transition from ice to water, but still leaves the actual process a mystery. 

Secondly, the transition between phases reflects processes which are not present at other times. Comparing ice and water droplets or ferromagnetic and paramagnetic states (veridical vision and hallucinations) does not directly indicate the process that takes one to the other. Thus, nucleation, the first step of self-organization in a phase transition from solid to liquid, can only be studied during melting. 

Additionally, within the critical transition region, processes fluctuate in response to their intrinsic dynamics, leading to finite durations which may map onto the transient phenomena seen in network models of hallucinations in, for example, Lewy body disorders [21].

Finally, comparing first- and second-order transitions puts a focus on how little we currently know of the phenomenology of transitions between veridical and hallucinatory vision. In contrast with first-order transitions, those of the second order occur without a discontinuous shift in physical properties. Whether transitions are abrupt or gradual may suggest which analogy is more apt. 

Our analogy therefore suggests that it will be productive to identify novel processes which are associated with hallucinations, and to focus investigations on the values of factors just around the time of transition. 

### 1.6. Evidence on the Role of Information Transfer from the Environment in Triggering Hallucinations

What might be a novel process, akin to nucleation, that starts the transition between veridical and hallucinatory vision? 

Reviewing the evidence above indicates that the predominant paradigm has been to treat the brain as the sole thing that needs to be understood if we are to model hallucinations. Generative models in particular suggest that it is when the brain is cut off from external information that hallucinations are more likely.

However, the brain does not function as a closed system, as there is a continual flow of information associated with both energy and material transfer between it and the environment [16]. Indeed, veridical perception is defined by the relationship between perception and that environment, and hallucination is defined by a different relationship. 

**Figure 6:**
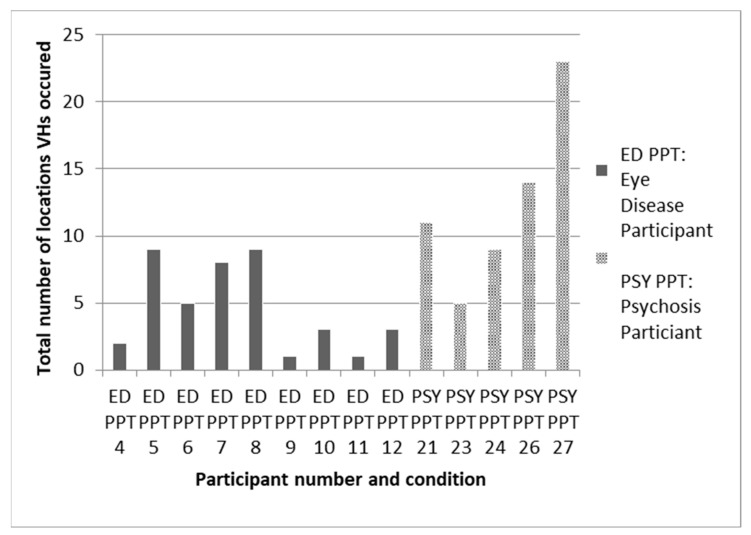
Data from daily diaries collected over a one-month period on total number of locations that participants with either eye disease (ED) or psychosis (PSY) recorded visual hallucinations (VH). Taken from Hannant, 2016 [23].

The granular relationship of the environment to hallucinations is poorly understood. What evidence there is, though mainly anecdotal, does suggest regularities in when and where hallucinatory episodes occur [22]. Thus, hallucinations are classically said to occur in a limited number of settings and to be more common at some times of the day than others. In support of this view, Figure 6 is a rare example of systematic data from a small study on eye disease and psychosis which shows that the majority of participants experienced hallucinations in fewer than 10 locations. 

Further evidence, some of it older and some very new, supports the possibility that particular information transfer is relevant to understanding how episodic hallucinations occur. However, it is limited and drawn from treatment studies that did not specifically focus on direct environment–hallucination links.

**Figure 7:**
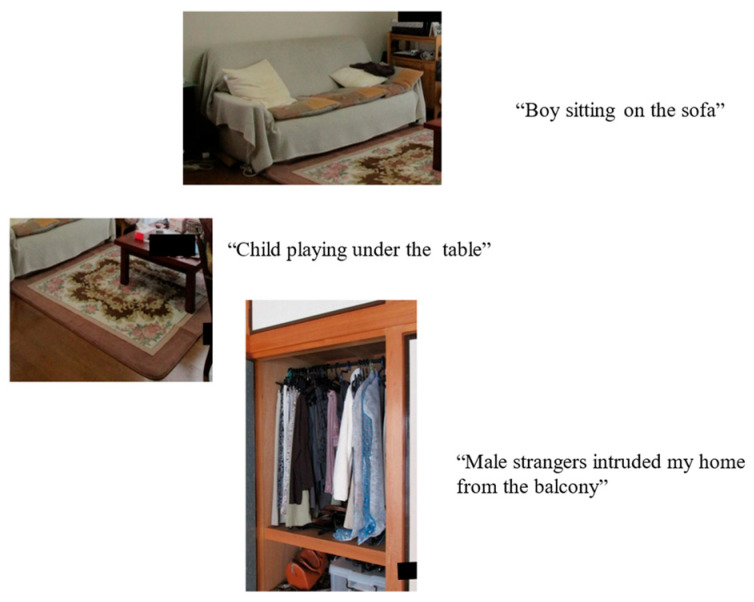
Mapping from environment to perception from Ishimaru et al. [24]. The pictures are of the objects which were associated with specific hallucinations and the text is a description of each hallucination.

Thus, two case studies recently reported by Ishimaru and colleagues [24] showed potential relationships between specific items in the environment and particular visual hallucinations. Removing that item mitigated the hallucination. They reported cushions on a settee triggering hallucinations of a boy, patterns on a carpet, hallucinations of children and hanging clothes, a vision of a person (Figure 7). This suggests several one-to-one relationships between trigger objects and hallucinations. There was not the normal one-to-one mapping, but a new one. This was not consistent (or stationary), however, since sometimes the trigger object was seen correctly. Where others would see a cushion, the hallucinator saw a boy, where others saw clothes, a person, and a pattern on a carpet, children. To return to Figure 1, there is the possibility not that the person is seen instead of the cushion and lamp, but that the cushion and lamp are seen as a person.

Turning to offset, twenty years ago, Diederich et al. [25] surveyed people who manage their own hallucinations and found that changes in the visual environment such as turning on a light or looking away can be effective in restoring veridical vision.

## 2. Re-Conceptualizing Hallucinatory Episodes

### 2.1. Onset of a Hallucinatory Episode—How Something Different Is Seen to What Is There

If hallucinations are specific to particular features of the environment, are generative models sufficient to account for hallucination onset, or is a different process, analogous to nucleation, required?

**Figure 8:**
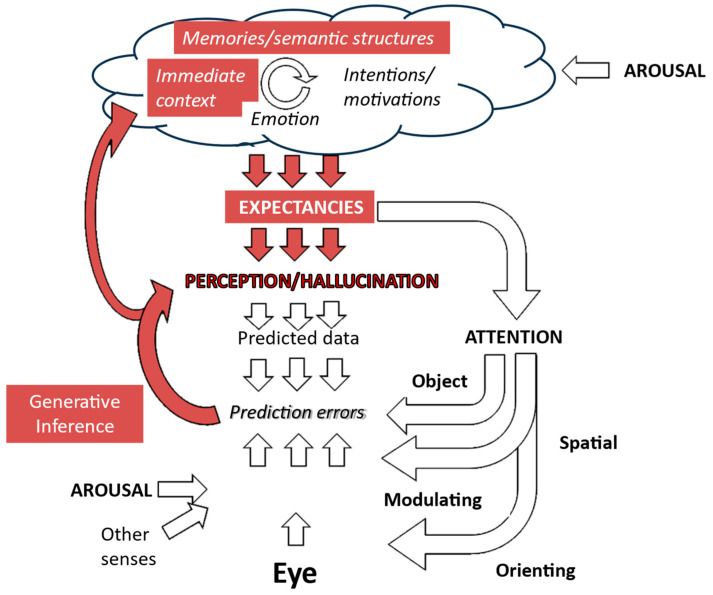
Generative inference and context effects. Preceding perceptions create a visual context which, in combination with memories of previous hallucinations, leads to heightened expectancies of that hallucination recurring which are not corrected to veridical perception because of a lack of effective prediction errors due either to sensory data being absent or disregarded. Figure adapted from Collerton et al., 2023 [3].

Previous models of hallucinations have suggested that these regularities are due to a general effect of visual and temporal context. These factors are potentially modulated by internal factors such as diurnal fluctuations in arousal and alertness, as illustrated in Figure 8. Thus, Active Inference would propose that a particular image of a child would be so highly expected (the representation so precise) in that temporal and spatial context that the child representation over-rides possibly degraded sensory data derived from a cushion at that particular point in that particular place. 

How plausible is this explanation? Direct evidence is lacking. There are data that people who hallucinate tend to see objects in meaningless noise (pareidolia) more [26], suggesting a general increased bias towards expectations influencing perception when sensory data are limited. And the more that a hallucination occurs in a specific context, the more that it would be expected, suggesting that specific expectancies could be learnt. However, generative models do not obviously account for the first occurrence of a hallucination when there should not be any high expectation to see that specific perception. Furthermore, the finding that removing the cushion significantly reduces the frequency of hallucination heightens the possibility that there is a specific one-to-one mapping between the cushion and the hallucination, not a more general link between the visual context and the hallucination, and not a lack of sensory data. If that latter were the case, then removing the cushion would have little effect on the occurrence of the hallucination.

We therefore suggest that the current balance of evidence (such as it is) means that, in at least some cases, hallucinatory episodes are potentially triggered and ended by specific information transfer from the environment. This raises the possibility that, as far as the perceptual system is concerned, the hallucination is not due to a lack of sensory data, but that it is a response to a specific pattern of information. To put it another way, there is an idiosyncratic perception of an object—something is seen, but not what other people would see as that object. 

How might it be that a cushion is seen as a boy or hanging clothes as a person? One possibility is that the trigger object looks so much like the hallucinated perception, that what is seen is a misperception or illusion rather than a true hallucination. This is not impossible. For example, one of Ishimura’s participants saw hanging clothes as a figure, an obvious overlap in form. However, we suggest that this is not the whole explanation, given that there is no obvious overlap in form between the triggers and hallucinations of the boy and children (Figure 7).

The alternative, then, is that for the hallucinator the cushion genuinely looks like a boy for some of the time. That is, that the sensory data derived from the cushion lead to an internal representation of a boy. Generative inference does not provide an obvious mechanism for this link.

However, this mapping may be understandable within the framework provided by another form of inference which the brain may also be capable of exhibiting—discriminative inference.

**Figure 9:**
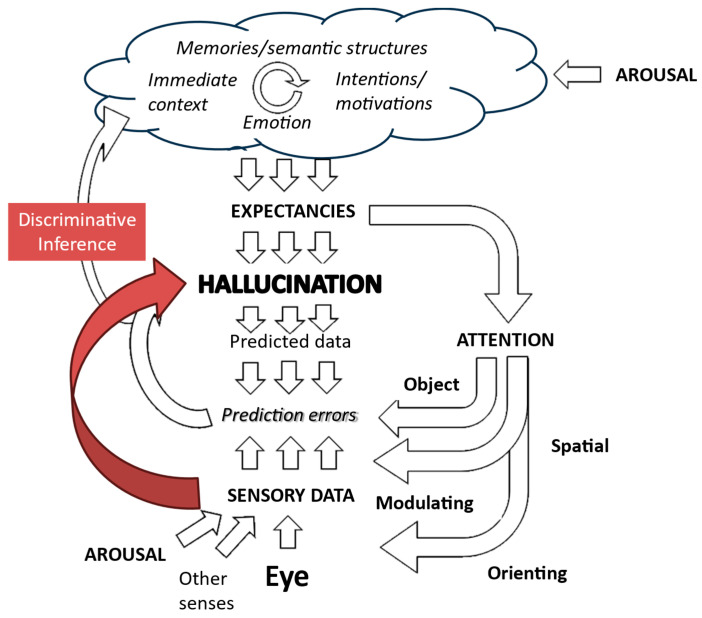
Putative role of aberrant discriminative inference in creating hallucinations. There is a direct link between the sensory data associated with a triggering object in the environment and the consequent hallucinatory perception. Figure adapted from Collerton et al., 2023 [3].

Discriminative inference is another model of how the brain links sensory data to internal representations [27]. It can be conceptualized as an inversion of generative approaches. Generative discrimination infers sensory data on the basis of expected perceptions, while discriminative inference infers perceptions on the basis of sensory data. Thus, it “emphasizes bottom-up signal flow, describing vision as a largely feedforward, discriminative inference process that filters and transforms the visual information to remove irrelevant variation and represent behaviorally relevant information in a format suitable for downstream functions of cognition and behavioral control. In this conception, vision is driven by the sensory data, and perception is direct because the processing proceeds from the data to the latent variables of interest” (Figure 9). Whether these two forms of inference reflect the functioning of different brain systems or different modes of functioning of common systems is not established. However, they are commonly modeled using different algorithms, exhibit different computational efficiencies in various scenarios, and show different strengths in accounting for perceptual phenomena. Taken together, these contrasts imply that the distinction between these types of inference has heuristic value in drawing attention to different ways in which perceptual systems may be functioning at different stages of hallucinations.

Discriminative inference has properties which make it attractive in explaining the link between a specific object and a hallucination. In theory, discriminative inference can lead to a direct link between any sensory input and any perception. These perceptions are much less bound by wider context than generative models propose. Furthermore, once a link is established, as far as the brain is concerned, that representation is what is really out there, and is relatively impervious to change. Additionally, discriminative inference supports one- or few-shot learning, meaning that a reliable link could be established from a single experience of a hallucination. Discriminative inference could form our analogy with nucleation in phase transitions.

However, this still leaves the question of how that link is established in the first place (as is also the case, however, with generative inference). Here we have to hypothesize. Several propositions are required. If we accept that the brain has a strong bias towards seeing something meaningful, as pareidolia would suggest, and that because of impairments in the visual system it cannot see what is there, as high rates of agnosia suggest [28], then it must see the least unlikely object that it can [29]. And in this case, based upon the setting and the characteristics of the cushion, the least unlikely thing to see is a boy. Though this conclusion is rather a leap, it does produce testable predictions of relationships between objects and perceptions in people prone to hallucinations.

Accepting that the link between trigger and hallucination, especially before hallucinations are well established, indicates that the brain is predominantly using discriminative rather than generative inference at that point raises the question of why discriminative inference predominates at that time. The current answer is that we do not know. However, our analogy with phase transitions may suggest why we do not know, in that the transition from solids to liquids occurs only within a very limited range of combined values of relevant variables such as temperature, pressure, and purity. Within that limited range, new processes such as nucleation occur. By analogy, the relative infrequency of hallucinatory perception may suggest that a particular infrequently occurring combination of factors within a limited range of values induces aberrant discriminative inference and a hallucination. Since we have not observed inference under those conditions, we cannot yet determine relevant factors. 

### 2.2. Types of Inference and Variational Principles

In Table 1, we highlight the key differences we have discussed between the potential roles of discriminative and generative inference in hallucinations as well as two associated factors, Shannon surprise and nonstationary systems, which may further tilt the balance towards the relevance of discriminative inference in hallucination onset. 

If it is the case that the brain is using a different form of inference when hallucinations are triggered, what might be the conditions under which that form of inference predominates. Consequently, what is the variational principle best suited to modelling it? Is minimization of free energy still the most compelling analogy, or would another principle better describe the phenomena at hand [30,31]? 

In this section, we explore some potential extensions of the free energy formulation as applied to hallucinations and suggest alternative approaches to supplement its undoubted strengths.

#### 2.2.1. Shannon Surprise and Discriminative Inference

In information theory, free energy is defined as a combination of Kullback–Leibler (KL) divergence and Shannon surprise. K-L divergence is a measure of the separation of two probability distributions, which in the case of Active Inference are predicted and actual sensory data. Shannon surprise is an estimate of how unlikely a particular environmental event might be. Friston argues persuasively that as organisms learn about their environment, they construct their generative models of the world and act on those such as to maximize the predictability of what happens to them and hence to minimize surprise [16]. As a consequence, Shannon surprise can be neglected in most cases. Thus, free energy becomes equivalent to KL divergence alone, and can be restated as a form of Bayesian inference (See Appendix A for further details).

However, in the case of hallucinatory episodes, Shannon surprise may be significant, at least at onset. At these points, there is a distinct discontinuity between the preceding veridical perception and the hallucination such that the hallucination cannot be predicted from the normal relationship between the world model and the environment. 

Since the Shannon surprise is significant, Bayesian inference with its basis in transitions from prior to posterior probability distributions is not relevant, and another process may look like a more attractive explanation. Since discriminative inference is specialized to rapidly categorize unexpected but behaviorally relevant information, it is a good candidate process to supplement generative inference in highly surprising circumstances. Indeed, significant Shannon surprise may be a general signal that the brain is shifting to discriminative inference in that instance. 

#### 2.2.2. Variational Principles for Nonstationary Brain Dynamics

We have suggested that the mapping between an object in the environment and perception is a key factor in modelling transitions between veridical and hallucinatory vision. In veridical vision, this relationship is stationary—it does not vary over time, nor does it change from observer to observer. Bayesian inference is a powerful approach for modelling stationary systems. However, hallucinatory episodes are by their nature nonstationary. They are not stable over time nor, probably, between observers. Thus, nonstationary variational principles are needed to model these phenomena. Nonstationary brain dynamics are highly complex and transitory when interacting with environments, thereby yielding intricate transitions between various “quasi attractors” or semi-stable states [32]. 

Several potential methods have therefore been proposed to deal with nonstationary states. We will highlight one, “self-organization with constraints” [33,34], which was developed as a guiding variational principle to overcome non-stationarity in open boundary systems. 

Constraints act upon a whole system such as a brain so that elementary dynamics can be self-organized to yield functional differentiations even under nonstationary dynamics [35]. Constraints can be intentional but quantitative, such as the maximization of mutual information or minimization of consumed energy. Although such constraints might appear at first glance to provide a top-down signal, in practice processing of bottom-up signals become predominant, since information contained in input signals facilitates functional differentiation for each elementary dynamical system. Thus, the predominant direction of information flow under nonstationary open boundary conditions is more like that proposed for discriminative inference than that for generative inference. 

In summary, we suggest that discriminative inference, significant Shannon surprise, non-stationarity, and self-organization with constraints provide an alternative approach to modelling the onset of hallucinatory episodes. 

### 2.3. Persistence of Hallucinations

If discriminative inference is relevant in the onset of a hallucination, might it also account for the persistence of hallucinations? Here, there is a less convincing argument that generative inference needs supplementing.

As we noted in Section 1.1, the persistence of hallucinations means that a key feature of generative models, responsiveness to prediction errors, is absent, otherwise the hallucination would disappear almost before it was experienced. Generative models can account for this absence by a combination of a high expectation of seeing (or continuing to see) the hallucination, and a restricted impact of sensory data. The presence of the hallucination in itself might plausibly suggest that the impacts of these two factors are intensified. Hence, a hallucinatory perception could both increase the expectation for that perception to occur, and shift attention from disconfirming data, given that attention is focused on the hallucinatory perception. Thus, Active Inference alone may be sufficient to account for the persistence of a hallucinatory episode.

### 2.4. Ending of Hallucination

Both forms of inference can provide an explanation for why hallucinations end. For Active Inference, once the representation is activated, and becomes over-valued, it is not challenged by sensory data until these sensory data become so inconsistent that the data mismatch over-rides the representation. For discriminative inference, once the trigger looks different enough, then the correspondence it has with hallucinations becomes sufficiently distant that another perception is inferred. The observation from Ishimaru et al. [24] that sometimes the cushion triggers the perception of a cushion and sometimes that of a boy together with Diederich et al.’s [25] findings that changing illumination alone can stop a hallucination suggest that changing the visual information coming into the brain in quite subtle ways is sufficient to initiate or abolish a hallucination even if the trigger object remains. This argues for a highly specific link between information coming from the object and the associated hallucinatory representation which needs to be maintained for the hallucination to continue. 

The role of highly specific information may point towards the contributions of specific perceptual systems in different stages of hallucinations. For example, luminosity information is relayed by the magnocellular visual system [36]. That turning on lights reduces hallucinations suggests that data flow via this pathway can reset vision towards veridicality.

### 2.5. Momentary Non-Equilibrium Thermodynamic Processes, Free Energy and the Transience of Hallucinations

Thus far, we have explored the potential roles of specific factors in triggering or abolishing hallucinatory episodes. In this final section, we will briefly raise the possible contribution of the thermodynamics of brain processes [37] to these phenomena. 

As used in information theory, free energy is not the same concept as thermodynamic free energy, though their mathematical treatment is equivalent under some conditions. We show in Appendix A how the treatment of thermodynamic free energy is a more general formulation of that for informational free energy. 

Energy consumption is associated with any information processing in the brain. Prompted by our analogy with phase transitions in which thermodynamic free energy is key, there may be useful questions to be raised not only of the role of energetic considerations in hallucinatory transitions, but also of how intrinsic brain dynamics are related to the finite duration of hallucinatory episodes. 

A null hypothesis would be that the energetic costs of veridical and hallucinatory vision are not different. However, as we note in Section 2.1, discriminative and generative inference have varying computational costs in different scenarios. Taken with hallucinatory episodes being both rare and transitory, this raises the possibility that energetic factors are relevant. However, defining and measuring these factors is challenging. 

## 3. Future Research Directions

Our analogy with phase transitions strongly suggests that relevant evidence to constrain possible models enough to make them testable needs direct observations of transitions between veridical and hallucinatory perception.

Ideally, there should be a small number of critical variables to investigate akin to temperature, pressure, or purity. However, brain systems are far more complex than solids or liquids, with an almost infinite number of potential variables which would be impossible to measure or model. We need to reduce the combinatorial explosion that brain complexity produces to a tractable level. Hence, temperature or other phase transition variables map onto complex combinations of brain factors. 

Consideration of the Consensus Framework as it applies to discriminative and generative inference (Figure 7 and Figure 9) can provisionally identify a limited number of variables which are directly linked to the hallucinatory representation. Hence, sensory data and attentional modulation of already gathered sensory data, together with expectations of specific perceptual representations, are key factors. Other functions such as context or orienting attention have indirect effects via these factors and so are of secondary importance. Focusing on these three key variables with algorithms aimed at either minimizing free energy or maximizing shared information should produce testable models.

However, there is a further complication in that there is no obvious way in which these cognitive functions could be observed during hallucinatory transitions. While there are tasks to measure attentional or perceptual performance, they would both affect and be affected by hallucinations. Therefore, studies may need to look at putative underlying brain functions, though this in itself introduces a further potentially unreliable mapping from cognitive to brain function [27]. A focus on brain function might, however, usefully limit relevant variables. For example, if as proposed in Section 2.4, specific brain pathways are influential in particular stages of hallucinatory episodes, identifying these as regions of interest would increase the power of analyses.

Additionally, observing these potential brain transitions is extremely challenging, given limitations in current technologies and methodologies. The responsiveness of hallucinations to the environment means that to actively investigate them is often to change or abolish them. Finding participants who can be observed during transitions, though not impossible, is quite a hurdle to overcome [38]. Furthermore, the timescales over which these transitions appear to occur (though very poorly documented), are shorter than those at which imaging technologies operate.

Nevertheless, there are some areas which could be profitably investigated at present. 

One is the phenomenology of transitions. Are these unitary phenomena or of heterogenous types, abrupt or gradual, smooth or irregular, directly from one veridical object to a corresponding hallucinatory object or via intermediate forms? Knowing what to account for in modelling transitions will constrain potential explanations and direct theories towards the most productive analogies. 

A second area would be to systematically explore the link between the environment and hallucinations, building on the work of Ishimaru and Diederich and colleagues [24,25]. Varying environmental factors to assess their impact on the onset and offset of hallucinatory episodes will clarify the relationship of information transfer from the environment to hallucinatory representations. 

Inferential models and their instantiation by network algorithms are learning models which adapt over time as new information is accrued. Hallucinations are also known to change over time, and it seems likely that the mechanisms that create them may also vary to account for these changes. We have proposed that the factors relevant in an initial hallucination may be different from those active in a well-established hallucination. Single hallucinations are a not infrequent experience for much of the non-clinical population (around 20% in last month [39]), suggesting that relatively random fluctuations in perceptual systems may lead to one instance of hallucinating. However, repeated hallucinations suggest systematic biases are active, such as increased expectancies, degraded perception, or robust linkages between specific environments and hallucinations. Investigating hallucinations at different stages of “maturity” may therefore be another means of testing the relative contributions of diverse factors.

## 4. Conclusions

We have explored the application of generative models and the free energy principle to explain how hallucinations are episodic. Our conclusion is that, as for all of the current approaches, the ability of generative models to account for why hallucinations occur at only some times and in only some places is underspecified.

We suggest that generative approaches may have particular strengths in accounting for established hallucinations and the persistence of specific episodes. We raise the possibility that the action of another form of inference altogether, discriminative inference, may be more influential in early hallucinations, and in the onset and offset of episodes. We present some data that allow this possibility.

Primarily though, we highlight large gaps in our present knowledge of what we are trying to explain. In large part, this reflects a historic lack of focus on the episodic nature of hallucinations. We expect that as a greater amount of systematic evidence is gathered on hallucinatory transitions, models will be refined and be more able to be tested.

## Figures and Tables

**Table 1:** Comparison between discriminative and generative inference in relations to hallucinations.

Type of Inference	Discriminative	Generative
Role in veridical perception	Rapid recognition of behaviourally significant information for perception	Perception of complex, convolved scenes, and objects within them.
Relationship to environment	Associated with specific objects (e.g. cushion)	Associated with general context
Stage of hallucinatory episode it applies to	Onset and offset	Persistence
Information transfer from environment	Direct and specific	Indirect and less specific via context and error term
Information Framework	Self-organization under constraints (maximization of shared information)	Minimization of free energy (Kullback–Leibler divergence and Shannon surprise)
Mechanism of hallucination	Self-organization of potential perceptions to maximise shared information between distorted sensory information and perception.	Overly precise expectations (priors) combined with imprecise or absent sensory data.
Applicable system	Nonstationary open nonequilibrium system	Stationary open nonequilibrium system

## Appendix A

This Appendix provides a brief review with some notes on the free energy principle, based on Bayesian inference.

Let x be environmental (latent) variables, and y be sensory signals. In a generative model, we suppose that the human brain infers external phenomena indicated by x, from sensory signals, *y*. In the following, $p\left(A|B\right)$ indicates a conditional probability of the event A occurring, provided that the event B has occurred. p(A,B) indicates a joint probability of both events A and B occurring. p(A) indicates a marginal distribution of A.

Hence, the brain can create $p\left(x,y\right)$ (the generative model) with equality $p\left(x,y\right)=p\left(x|y\right)p\left(y\right),$ using the distribution of sensory signals. Adopting a Bayesian estimation represented by $p\left(x|y\right)=p\left(y|x\right)\frac{p\left(x\right)}{p\left(y\right)},$ this generative model can be written as $p\left(x,y\right)=p\left(y|x\right)p\left(x\right).$ In active inference, a prior probability is given by p(x), which implies an a priori belief, whereas $p\left(x|y\right)$ implies an a posteriori belief. For this latter, let q(x) be an approximate a posteriori probability distribution. Now, consider the difference between the two probability distributions in an informational sense, which can be represented by the Kullback–Leibler (KL) divergence, $D(q(x)∥p(x|y))=\int q(x)\log(\frac{q\left(x\right)}{p\left(x|y\right)})dx$ by definition. Then, we easily derive the following equation:

(A1) $$D(q\left(x\right)∥p\left(x|y\right))=\int q(x)\log\frac{q(x)}{p(x,y)}dx-(-\log p(y)).$$

It should be noted, however, that q(x) is absolutely continuous with respect to p(x|y), such that the Radon–Nikodym derivative dq(x)/dp(x|y) exists. This demands that a small change in approximate inferred results for external phenomena, which may cause a change in expectancies, follows a small change in a posteriori belief for these phenomena. This seems to be reasonable in the context of Active Inference, in which case we can use the above KL divergence.

Here, we assume that $p\left(x,y\right)$ follows Gibbs distribution, $e^{-U(x,y)/k_{B}T}$, where U(x,y) is internal energy, T is an absolute temperature, and $k_{B}$ is the Boltzmann constant. This assumption stems from Jaynes’ maximum entropy principle: one should take a probability distribution such as maximizing entropy when any appropriate distribution is not known [40]. Thus, internal energy takes the following expression:

(A2) $$U\left(x,y\right)=-k_{B}Tlogp\left(x,y\right)$$

(A3) $$=-k_{B}Tlogp\left(y|x\right)-k_{B}Tlogp(x).$$

Equation (A3) implies that the internal energy can be expressed by a summation of energy associated with perceiving signals and latent energy included in the latent variable.

Taking an average of internal energy with an approximate a posteriori probability distribution q(x), and using a formula that [free energy] equals [averaged internal energy] minus ([temperature] times [entropy]) in the sense of Helmholtz, free energy can be expressed in the following way.

(A4) $$F\left(q,p;y\right)=\int q\left(x\right)U\left(x,y\right)dx-(-T\int q\left(x\right)\log q\left(x\right)dx)$$

(A5) $$=-k_{B}T\int q\left(x\right)\log p\left(y|x\right)dx-k_{B}T\int q\left(x\right)\log p\left(x\right)dx+T\int q\left(x\right)\log q\left(x\right)dx$$

(A6) $$=k_{B}T\int q\left(x\right)log\frac{p\left(x\right)}{p\left(x,y\right)}dx-k_{B}T\int q\left(x\right)\log p\left(x\right)dx-T(-\int q\left(x\right)\log q\left(x\right)dx)$$

(A7) $$=k_{B}T\int q\left(x\right)\log\frac{1}{p(x,y)}dx-T(-\int q\left(x\right)\log q\left(x\right)dx).$$

Equation (A5) comes from the expression (A3) of internal energy. This implies that free energy here is interpreted as energy necessary only for perception independent of approximate a posteriori belief.

Equations (A6) and (A7) are given in order so that their relationship to Bayesian inference can be seen. It should be noted that Equation (A7) is straightforwardly derived from the expression of internal energy, Equation (A2) and the definition of free energy.

From Equations (A1) and (A7), in the case of $k_{B}=1$ and T=1, Friston’s formula $D(q\left(x\right)∥p\left(x|y\right))=F\left(q,p;y\right)-(-\log p(y))\;is\;derived,\;as\;F\left(q,p;y\right)\;becomes\;equal\;to\int q\left(x\right)log\frac{q\left(x\right)}{p\left(x,y\right)}dx.$ The second term, −log⁡p(y), is called a Shannon surprise. If a sensory signal does not change, the variational principle obeys the requirement of a free energy minimum. However, drawing an inference about the environment by a living organism also demands changing sensory signals in an active inference. This leads to the additional requirement of minimizing a Shannon surprise. Then, a variation should be taken for a whole of KL divergence.

The assumption that $k_{B}=1$ and T=1 is allowed if we think of information contents only. However, if we consider the energetics associated with information processing, which are important in biological, particularly neural systems, by inquiring how much energy is necessary for the concerned information processing, the following formula should be taken into account:

(A8) $$D\left(q\left(x\right)∥p\left(x|y\right)\right)=F\left(q,p:y\right)/k_{B}T-(1/k_{B}-1)\int q(x)\log q\left(x\right)dx-(-\log p(y)).$$

In order to show the biological significance of the Boltzmann constant and real values of absolute temperature, let us consider, for example, amount of energy necessary for reproduction of nucleotide, which can be estimated by $k_{B}N_{A}T\log\frac{g}{\delta }$ in the formulation as birth and death process, where g indicates a generation rate, δ a decay rate, $k_{B}$ Boltzmann constant, 1.38 × $10^{-23}J/K$, $N_{A}$ Avogadro constant, 6.02 × $10^{23}/mol$, and T absolute temperature [41]. According to the estimation by England, one can obtain the following energy amount estimated as amount of heat released for reproduction: for T=300 K, 6.2 kcal/mol in case of RNA, while 15.6 kcal/mol in case of DNA, which exceeds an allowed value by thermodynamics, 10 kcal/mol. This implies that DNA was organized to use much more entropy production than RNA, such as maintaining information contents of the gene following RNA’s reproduction speed [41].

Although this is an application at the microscopic level, both the Boltzmann constant and absolute temperature are important even at the macroscopic levels of information processing associated with the mass dynamics of neural networks and consequent energetics, such as the dynamic change in the activity of neural networks to reflect different information contents, for example, between veridical and hallucinatory perception.
